# Patterns in *Leptospira* Shedding in Norway Rats (*Rattus norvegicus*) from Brazilian Slum Communities at High Risk of Disease Transmission

**DOI:** 10.1371/journal.pntd.0003819

**Published:** 2015-06-05

**Authors:** Federico Costa, Elsio A. Wunder, Daiana De Oliveira, Vimla Bisht, Gorete Rodrigues, Mitermayer G. Reis, Albert I. Ko, Mike Begon, James E. Childs

**Affiliations:** 1 Centro de Pesquisas Gonçalo Moniz, Fundação Oswaldo Cruz, Ministério da Saúde, Salvador, Brazil; 2 Instituto de Saúde Coletiva, Universidade Federal da Bahia (UFBA), Salvador, Brazil; 3 Department of Epidemiology of Microbial Disease, Yale School of Public Health, New Haven, Connecticut, United States of America; 4 Institute of Integrative Biology, University of Liverpool, Liverpool, United Kingdom; 5 Centro de Controle de Zoonoses, Secretaria Municipal de Saúde, Ministério da Saúde, Salvador, Brazil; University of Tennessee, UNITED STATES

## Abstract

**Background:**

We address some critical but unknown parameters of individuals and populations of Norway rats (*Rattus norvegicus*) that influence leptospiral infection, maintenance and spirochetal loads shed in urine, which contaminates the environment ultimately leading to human infection.

**Methodology/Principal Findings:**

Our study, conducted in Salvador, Brazil, established the average load of leptospires in positive kidneys to be 5.9 x 10^6^ per mL (range 3.1-8.2 x10^6^) genome equivalents (GEq), similar to the 6.1 x 10^6^ per ml (range 2.2-9.4 x10^6^) average obtained from paired urines, with a significant positive correlation (R^2^=0.78) between the two. Based on bivariate and multivariate modeling, we found with both kidney and urine samples that leptospiral loads increased with the age of rats (based on the index of body length to mass), MAT titer and the presence of wounding/scars, and varied with site of capture. Some associations were modified by sex but trends were apparent. Combining with data on the demographic properties and prevalence of leptospiral carriage in rat populations in Salvador, we estimated that daily leptospiral loads shed in the urine of a population of 82 individuals exceeded 9.1 x 10^10^ leptospires.

**Conclusions/Significance:**

These factors directly influence the risk of leptospiral acquisition among humans and provide essential epidemiological information linking properties of rat populations with risk of human infection.

## Author Summary

Leptospirosis is a human disease caused by the bacterium *Leptospira*. It is often acquired through contact with water/soil contaminated with leptospires shed in the urine of rats, the most important reservoirs in urban environment. We evaluated how location, sex, age and other rat characteristics can influence the amount of leptospires shed into the environment. We found that rats from different locations were able to shed different concentrations of leptospires. Rat populations were able to shed more than one billion leptospires per day. The findings of this study provide information linking rat population and the risk of human leptospirosis.

## Introduction

Leptospirosis is a human disease caused by a spirochete of the genus *Leptospira* and is most often acquired through contact with environments contaminated with leptospires shed in the urine of infected reservoir mammalian hosts. Leptospirosis is a global public health problem affecting rural and urban populations of both developed and developing nations [1–4]. The estimated annual incidence of leptospirosis exceeds 1 million cases, with a mortality of approximately 10% [5,6]. The increasing recognition of acute renal failure and pulmonary hemorrhagic syndrome accompanying leptospirosis has prompted the WHO to call for increasing surveillance to more accurately determine the global burden of leptospirosis, to increase awareness of the disease in developing countries, and to improve the methods and standards of disease surveillance and control [5]. Pulmonary hemorrhagic syndrome is characterized by massive pulmonary bleeding and acute respiratory distress, and is now reported worldwide and associated with a case fatality of >50% [2,7,8].

In Brazil, as in other tropical developing countries, slum dwellers are at high risk for leptospirosis due to limited access to health care and poor sanitary conditions within neighborhoods [2]. Accumulations of uncollected refuse, the presence of open sewers, and the poor construction of residences create conditions conducive to supporting large populations of rats (the Norway rat, *Rattus norvegicus* and/or the black rat, *Rattus rattus*), which are the primary reservoir hosts of leptospires transmitted to humans in urban locations [9–21]. Direct contact with infected rats, or contact with water and mud contaminated with *Leptospira* spp. shed in the urine of rats, are the primary routes of transmission in these settings. Predictable seasonal spikes in leptospirosis incidence in large urban centers of Brazil are associated with heavy rainfall during the winter months [2,22,23].

Although leptospirosis is caused by many pathogenic species in the genus, the Icterohaemorrhagiae complex causes the most severe disease and is closely associated with both Norway rats and the black rat [20,21,24]. Herein, we restrict our comments to this leptospiral complex and the critical role of the Norway rat in leptospiral transmission to humans, as this species is the primary reservoir host for leptospires within Salvador, Brazil, where our work was carried out [14,15,17].

Norway rats are frequently infected with leptospires in both tropical and temperate cities. For example, five studies published during the period 2003–2014 report the prevalence of leptospiral infection among urban rats to range between 11.1% (N = 592) in Vancouver, Canada [18], 16% (N = 127) in Tokyo, Japan [20], 48% (N = 23) in Santa Fe, Argentina [25], 65.3% (N = 201) in Baltimore, USA [19], and 63% to 83% (N = 226) in Salvador, Brazil [14]. However, the methods used to determine infection varied, including combinations of PCR-based detection of DNA from rat kidney samples, serum antibody detection, and isolation or antigen detection of leptospires in kidney, so direct comparison of these results is precluded.

Moreover, reports from this geographical range of leptospiral carriage among urban Norway rat populations do not provide information on a critical epidemiological parameters that directly link the load of leptospires shed in urine to the degree of environmental contamination that ultimately determines the risk of transmission to humans. Once infected, Norway rats establish a chronic carriage state with leptospires residing within the proximal tubules of kidneys. Norway rats are considered asymptomatic carriers, as evidence of leptospiral-induced pathology is minimal [26] and experimentally infected rats gain weight at the same rate as non-infected controls [27]. Infected rats have the ability to shed viable leptospires in their urine throughout their lives [26,28,29]. The few studies documenting the load of leptospires shed in the urine infected Norway rats are based on experimental studies; they report loads as high as 10^7^ genome equivalents (GEq)/ml, as measured by quantitative PCR (qPCR) or dark field microscopy [27,30], with peak levels of shedding being reached within 28 days.

The presence of serum antibodies does not indicate clearance of leptospires from the kidney [21], but its potential influence on the load of leptospires shed in rat urine is unknown, Once leptospires are shed into the environment, they can survive from days to months [31,32]. However, given the inherent variability of the environment (e.g. soil type, water content, microbiome present, pH etc.), these estimates require validation under conditions representative at field sites.

In this report we examine some of the critical, but unknown, parameters that influence the role of individual rats (and hence rat populations) in maintaining leptospiral infection and that contribute to the urine load of bacteria shed into the environment. Specifically, we describe how the location, sex, age and presence of serum antibody in individual rats influence leptospiral carriage and urine shedding loads and how these parameter estimates, when applied to the demographic characteristics of a previously described [33] rat population in Salvador, determine the degree of environmental contamination potentially influencing the risk of human infection.

## Methods

### Study sites and data collection

The study sites, data collection methods and types of samples obtained for this study have been described previously [14,33]. Briefly, during June-August of 2010, we captured Norway rats from five urban slum locations in Salvador, Brazil. Sites were selected based on the high annual incidence of severe human leptospirosis reported from these communities in 2010 [14,16]. Study sites were systematically sampled by setting three to five Tomahawk live traps at each of eight contiguous households [33]. Rats were euthanized and sex, weight and the presence of scars (based on a five point wound score) were recorded [14]. Mass has been shown to be excellent proxy for estimating rat age [33–35]. However, we also used the length to mass ratio (L/M) as an additional proxy [36].

Blood was obtained by cardiac puncture using a 5mL syringe, and serum was recovered after centrifugation. Urine was obtained directly from the bladder with a 1mL syringe after which the kidneys were removed. All samples, except kidney smears on slides, were immediately stored at -80°C until tested by qPCR to determine kidney and urine loads of leptospires and by microagglutination tests (MAT) to determine antibody titers. Sera testing positive by MAT at screening were diluted to obtain endpoint titers.

### Spiking experiments

We performed two spiking experiments, the first with water and the second using rat urine, to assess the quality of our extraction procedures and their accuracy in quantifying leptospiral loads. The appropriate amount of leptospires (*Leptospira interrogans* serovar Copenhageni strain Fiocruz L1-130 [37], hereafter abbreviated as strain L1-130) was spiked into 200μL of water to achieve a final concentration of 1 x 10^8^ leptospires/mL. The same process was performed using rat urine for the second experiment. Rat urine was obtained from an uninfected wild rat (no indication of kidney colonization by culture, and negative IFA and qPCR results for the presence of leptospiral antigen or DNA in kidney samples). After the spiking, serial 10-fold dilutions of 1 x 10^7^ to 1 x 10^0^ leptospires/mL were performed, using water and urine, for the first and second experiments respectively, as diluent. Spiking indicated a near perfect correlation between the number of spiked leptospires and the GEq detected by qPCR assay in urine (R^2^ = 0.9998) as well as water (R^2^ = 0.9997) (S1 Fig).

### Quantitative real-time PCR of *Leptospira* load in kidney and urine

DNA was prepared from 25mg and 200μL of previously frozen kidney and urine, respectively, with the automated Maxwell 16 System DNA Purification Kits (Promega Corp., Madison, WI). Quantitative real-time PCR (qPCR) for leptospirosis was performed using 5’ nuclease (TaqMan) assay and primers that amplified a sequence of *lipL32*, a gene that is exclusively present in pathogenic *Leptospira* [38]. For the calibration curve, genomic DNA obtained from strain L1-130 [37] was quantified using an ND-1000 spectrophotometer (Nanodrop Technologies, Wilmington, DE). Genomic equivalents were calculated based on a genome size of 4,627Mb [37]. Eight calibrators (10^0^ to 10^7^ GEq/mL) were prepared upon adjustment of DNA concentration to 10^7^ GEq/reaction followed by ten-fold serial dilutions. Quantitative real-time PCR amplifications were performed using an ABI 7500 Real-Time PCR System (Applied Biosystems, Foster, CA). PCR conditions were adapted from a previously described method [38]. The reaction mix consisted of 12.5μL of Platinum Quantitative PCR SuperMix-UDG (Invitrogen, Carlsbad, CA), 500nM of forward and reverse primers, 100nM of probe, 5μL of DNA extract and ultrapure water to a final volume of 25μL. The amplification protocol consisted of 2 min at 50°C and 10 min at 95°C, followed by 45 cycles of denaturation at 95°C for 15s and annealing/extension at 60°C for 1 min. As an internal control to monitor inhibition of PCR amplification and the efficiency of DNA extraction, we constructed primers to test the presence of a rodent housekeeping gene glyceraldehyde-3-phophate dehydrogenase (*gapdh*). The primer pair and probe were designed using Primer Express version 1.3 (Applied Biosystems). The forward primer of GAPDH_F (5’- GGT GGA GCC AAG AGG GTC AT-3’) and GAPDH_R (5’-GGT TCA CAC CCA TCA CAA ACA T-3’) were selected to amplify a fragment that was detected by the probe, GAPDH_P (FAM-5’- ATC TCC GCA CCT TCT GCT GAT GCC-3’-BHQ1). Samples were tested in duplicate, and no-template controls (5μL of ultrapure water added instead of DNA) were included in each run. Samples in which replicates were detected within 40 PCR cycles were considered positives. Positive samples were Sanger sequenced to confirm the amplification of the *lipL32* gene from *Leptospira* species.

### Microscopic agglutination test (MAT)

Using the Microscopic Agglutination Test (MAT) [39] we tested rat sera against L1-130 strain to determine antibody titers. Positive rat sera at the screening dilutions of 1:50 and 1:100 were titrated using two-fold dilutions to establish endpoint agglutination titers, defined as the highest dilution where 50% or more of the cells where agglutinated [39].

### Statistical analyses

We evaluated the associations of *Leptospira* load in both urine and kidney with sex, sexual maturity, mass, L/M ratios, MAT titer and site of capture. Leptospiral loads, as measured by GEq of leptospiral DNA per mg of kidney and mL of urine, were log transformed for all analyses. We used ANOVA, applying the Bonferroni correction method [40], to evaluate the effects for each variable on the leptospiral loads in kidney and urine. The Shapiro–Wilk test [41] confirmed that log GEq per ml values in both kidney and urine were log-normally distributed. Variables associated with *Leptospira* load in kidney with a p<0.1 where included in multivariate analysis (linear regression), and a backward elimination strategy and the Akaike information criteria (AIC) [42] were used to select the best adjusted model: from amongst models with and AIC of 2 of the lowest value, the simplest model was chosen on grounds of parsimony. The same strategy was used to develop a separate model to predict *Leptospira* load in urine.

We estimated the contribution of a demographically stratified rat population to the degree of environmental contamination through contaminated urine. We used demographic characteristics of the rat population considered for this study, which comprised 82 rats (16 rats <200g, 46 rats weighting between 201–400 and 20 rats >400g). We considered the number of rats (NR) at each weight class, their respective *Leptospira* prevalence as evaluated by qPCR (PREV), volume (in ml) of urine shed per 24h (VOL), and log of GEq/ml of urine (LOAD). So the amount of leptospire shed per day at each weight group was calculated as (NR*PREV*VOL*LOAD). Volume of urine shed per day based on rat mass was obtained from previous studies performed in laboratory conditions [43]. We also combined the results from the three weight groups to obtain the total number (GEq) of leptospires excreted by this population per day. Finally, we divided this estimate by the number of rats captured per m^2^ at each site to estimate the number of leptospires shed per day at a given density of rats (NR*PREV*VOL*LOAD) / m^2^. We repeated this procedure for each one of the five collection sites.

### Ethics statement

Institutional Animal Care and Use Committee (IACUC) from Brazil and United States approved all the protocols used in this study. At Oswaldo Cruz Foundation, Salvador, Brazil; the *Comissão de Ética no Uso de Animais (CEUA) do CPqGM-FIOCRUZ-BA* approved the protocol number 003/2012. At the United States the Yale University's Institutional Animal Care and Use Committee (IACUC), New Haven, Connecticut, approved the protocol number 2012–11498. Protocols adhered to PHS policy, USDA Regulations, the US National Research Council Guide for the Care and Use of Laboratory Animals and all US federal regulations.

## Results

A total of 82 *R*. *norvegicus* were captured from June to September 2010. Forty-five rats were captured from four sites from the Pau da Lima neighborhood (PL1, PL2, PL3 and PL8) and 37 from Sete de Abril neighborhood (7A). The demographic structure and *Leptospira* carriage prevalence of this population were described previously [14,33,44]. Demographic characteristics between sites were not different as described in S1 Table. Kidney and urine samples were obtained from 72 and 55 animals, respectively, of which 88% and 84%, respectively, were qPCR positive (Table 1). Five animals were negative for both kidney and urine and were excluded from further analyses of GEq titers, but were retained in analyses estimating the association of leptospiral loads in kidney and urine.

**Table 1:** Bivariate analyses of *Leptospira* load in kidney and urine of wild Norway rats.

	Kidney				Urine			
Characteristics	No.	PCR positive samples No. (%)	Mean Log_10_ PCR positive samples	SD^1^	No.	PCR positive samples No. (%)	Mean Log_10_ PCR positive samples	SD
Total	72	64 (88)	5.9	1.2	55	46 (84)	6.1	1.5
Sex								
Male	35	31 (88)	6.0	1.2	30	25 (83)	6.1	1.4
Female	37	33 (89)	5.9	1.2	25	21 (84)	5.9	1.7
Weight category								
Juvenile	15	11 (77)	5.6	1.1	7	5 (71)	4.9	1.9
Sub-adult	38	35 (92)	5.9	1.2	30	25 (83)	6.2	1.6
Adult	19	18 (95)	6.0	1.2	18	16 (89)	6.2	1.3
Ratio length/weight category								
I (>1.25)	13	9 (69)	5.6	1.2	6	4 (67)	**4.4** ^2^	1.8
II (0.85–1.25)	22	22 (100)	5.9	1.2	16	13 (81)	5.9	1.9
III (˂0.85)	37	33 (89)	6.1	1.2	33	29 (87)	**6.3**	1.2
Wounds/Scars								
0	29	25 (86)	5.6	1.2	20	14 (70)	**5.4**	1.7
1	26	24 (92)	6.1	1.0	18	17 (94)	5.9	1.5
≥2	17	15 (88)	6.4	1.2	17	15 (88)	**6.8**	1.3
Pregnant								
No	19	15 (79)	5.8	1.1	8	5 (62)	6.4	0.7
Yes	18	18 (100)	5.9	1.4	17	16 (94)	5.8	1.9
Vagina								
Closed	5	4 (80)	5.2	NA	2	1 (50)	5.2	NA
Open	32	29 (90)	5.2	2.6	23	20 (87)	6.0	1.7
Lactation								
No	34	30 (88)	5.8	1.2	24	20 (83)	6.1	1.7
Yes	3	3 (100)	6.4	1.2	1	1 (100)	4.2	NA
MAT Titer								
0	40	34 (85)	5.6	1.2	29	21 (72)	5.7	1.5
50	12	12 (100)	6.1	0.8	11	11 (100)	6.1	1.7
100	9	9 (100)	6.9	0.8	10	10 (100)	6.8	1.6
Site								
PL8	6	5 (83)	5.3	1.6	6	4 (67)	**4.5**	2.0
7A	32	26 (81)	5.9	1.2	18	14 (78)	5.6	1.3
PL2	14	14 (100)	5.4	0.9	11	11 (100)	6.1	1.4
PL6	5	5 (100)	6.4	1.3	5	5 (100)	6.0	2.1
PL1	15	14 (93)	6.6	0.9	13	12 (92)	**7.0**	1.0

^1^Standard Deviation

^2^Bold items reflect significant differences (P<0.05 ANOVA adjusted by Bonferroni correction method).

Values given are log10 genome equivalents per mm^3^ of kidney or per ml of urine.

**Fig 1:**
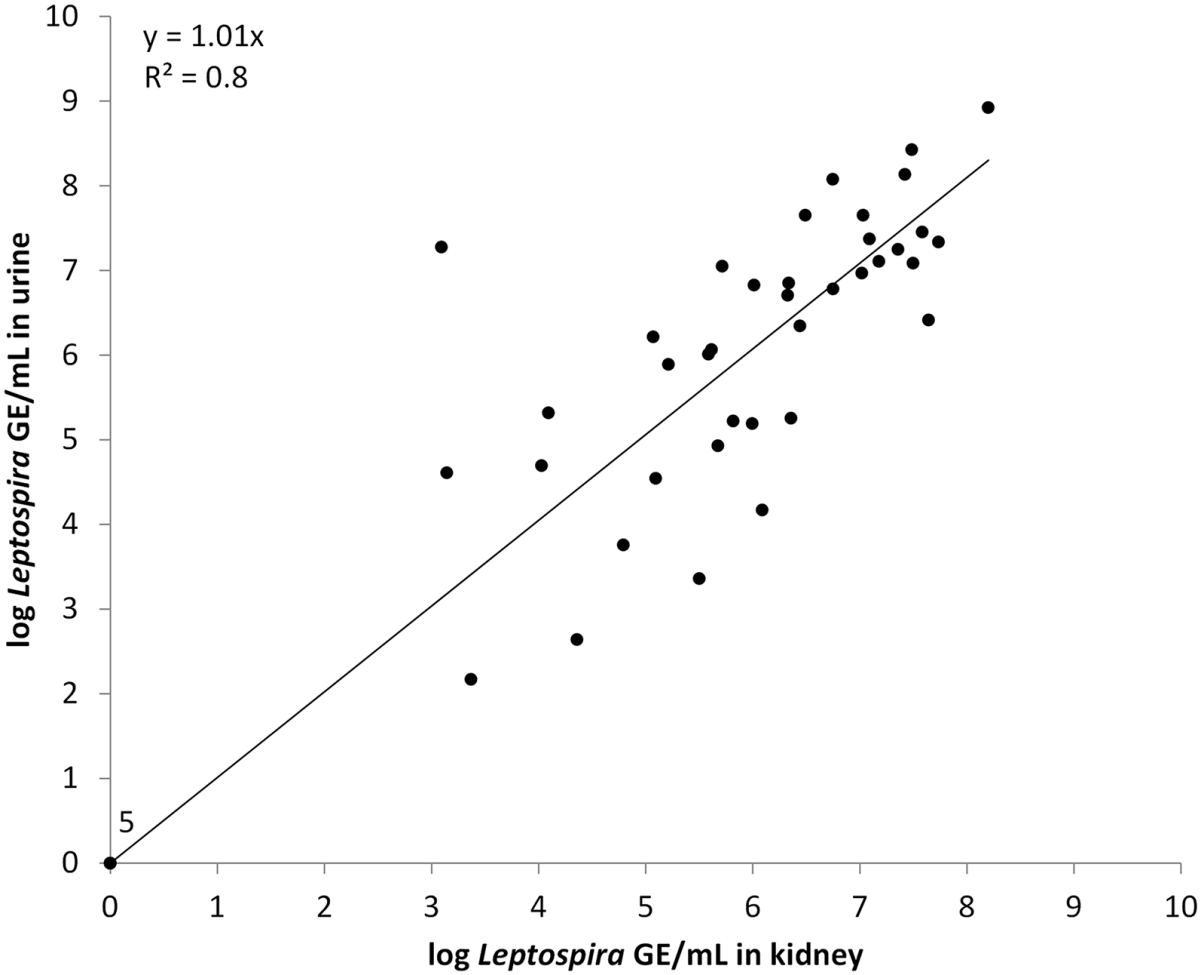
Correlation between the GEq load of leptospires in the paired kidney and urine samples of *Rattus norvegicus* from Brazil, 2010.

The average GEq of leptospires in positive kidneys was 5.9 x 10^6^, slightly lower than the 6.1 x 10^6^ average obtained from positive urines, not quite attaining statistical significance (p = 0.057). The range of GEq for positive kidneys and urines was 3.1–8.2 x10^6^ and 2.2–9.4 x10^6^, respectively (Table 1), and there was a strong and significant positive correlation (R^2^ = 0.78) between the GEq load of leptospires in the paired kidney and urine samples (Fig 1).

In bivariate analyses, leptospiral loads in kidney and urine tended to increase with W/L ratio (significant only in urine), increasing number of wounds/scars (significant only in urine), and varied with location of capture (PL1 vs. PL 8, significant only in urine) (Table 1). Male and female rats did not differ in the percentage of qPCR positive kidneys or urines, and their kidney and urine GEqs were indistinguishable. The reproductive status of females did not influence leptospiral loads (Table 1).

Separate multivariate linear regression models of GEq in kidney and urine included all the covariates for which at least one association had P<0.1 in bivariate comparisons (Table 1). In addition, any covariate that was retained in either the kidney or urine model was also included in the other model. Overall, the linear regression models from kidney and urine included the same set of variables with highly concordant results. In the kidney model, location of capture, MAT titer and the increasing number of wound/scars per animal were independent risk factors influencing GEq when all rats were pooled, though slight but significant differences in response were found between the sexes (although variables trended the same way; Table 2). In males, increasing W/L ratio was associated with increasing GEq, but not in females. Overall, increasing MAT titer was associated with greater GEq, but only in females. Similarly, increasing number of wounds/scars was associated with higher GEq, but only in females.

**Table 2:** Multivariate linear regression models of the relation between Leptospira load in kidneys and urine.

	Kidney			Urine		
Population	Total	Female	Male	Total	Females	Males
AIC	172.6	90.6	79.8^1^	147.3^2^	91.6^3^	72.7^4^
	Coefficient	(p value)		Coefficient	(p value)	
Ratio Length/Weight (continuous)			-2.02 (0.06)	**-1.71 (0.04)**		**-3.43 (0.01)**
Site PL8	**1.0 (ref)** ^5^		**1.0 (ref)**	**1.0 (ref)**	**1.0 (ref)**	1.0 (ref)
Site PL2	1.03 (0.12)		1.27 (0.22)	**1.80 (0.04)**	2.74 (0.07)	0.56 (0.60)
Site 7A	0.50 (0.36)		0.39 (0.64)	**1.90 (0.01)**	**3.05 (0.01)**	0.48 (0.59)
Site PL6	0.58 (0.26)		1.25 (0.06)	1.21 (0.10)	**3.12 (0.01)**	0.44 (0.64)
Site PL1	**1.12 (0.05)**		**1.81 (0.05)**	**2.80 (0.04)**	**3.86 (0.00)**	1.80 (0.07)
MAT 0	**1.0 (ref)**	**1.0 (ref)**	1.0 (ref)	1.0 (ref)	1.0 (ref)	1.0 (ref)
MAT 50	0.21 (0.58)	**1.01 (0.04)**	-0.58 (0.32)	0.07 (0.87)	1.55 (0.11)	-0.31 (0.64)
MAT ≥100	**0.86 (0.02)**	**1.61 (0.00)**	0.11 (0.88)	0.59 (0.26)	0.33 (0.65)	1.05 (0.19)
Scars 0	**1.0 (ref)**	**1.0 (ref)**		**1.0 (ref)**		
Scars 1	0.61 (0.06)	**0.91 (0.02)**		0.71 (0.15)		
Scars ≥2	**1.05 (0.01)**	**1.51 (0.01)**		**1.42 (0.01)**		

^1^AIC excluding MAT = 93.5

^2^AIC excluding MAT = 150.7

^3^AIC excluding MAT = 92.4

^4^AIC excluding MAT = 86.4

^5^Bold items reflect significant differences (P<0.05).

In the urine model W/L ratio, location of capture, and increasing number of wound/scars per animal were independent risk factors influencing GEq when all rats were pooled, but differences in response was found between the sexes (Table 2). Of note, including MAT titers increased the fit of the urine model, although not significant in bivariate comparisons. Males with higher W/L ratio had greater GEq in urine, but this association was not significant in females. Overall, three sites of rodent capture (PL2, 7A and PL1) produced animals with significantly higher urine leptospiral loads than the PL8 site (the reference and lowest value). Increasing number of wounds/scars was associated with higher GEq, but only when males and females were grouped.

Our estimate of the contribution of leptospires shed in the urine of a rat population of 82 individuals, stratified into three age classes, was 9.1 x 10^10^ per day with a mean density of 5.0 x 10^10^ per m^2^ of soil around households (Table 3). The independent estimates for rats captured at different sites showing the contribution of demography and leptospire load are shown in S2 Table.

**Table 3:** Estimates of Leptospira shedding loads in urine from 82 Norway rats based on properties of the demographic structure of the rat population from Salvador, Brazil.

	No. of rats^1^	Prevalence in urine^2^	ml/ day^3^	Log_10_ GEq/ml^4^	Log_10_ Leptospires shed per day^5^	Log_10_ Leptospires per m^2^ of soil /day
**Total**	**82**				**9.1**	**5.0**
Juvenile	16	0.77	15	5.6		
Sub-adult	46	0.92	20	5.9		
Adult	20	0.95	20	6		

^1^ Number of rats in each mass/age class (NR.)

^2^ *Leptospira* prevalence in kidney (PREV: Table 1).

^3^ Volume (ml) of urine shed per 24 hours (VOL) as described by Donaldson [43].

^4^ Genomic equivalents of *Leptospira* per ml (LOAD: Table 1).

^5^ Based on density of rats (DENS) captured around households.

## Discussion

We describe for the first time results that simultaneously investigated how differences between location of capture, sex, age (mass and W/L ratio indices), MAT markers of immune response and the prevalence of wounds/scars influence leptospiral loads of kidney carriage and associated loads shed in urine. Additionally, by extrapolating the values obtained in our analyses, we estimate the daily burden of leptospires shed into the environment by a rat population from an urban slum setting in Brazil [14].

The near perfect correlation between leptospiral loads in urine and kidney indicate that data obtained by measuring GEq in kidney samples alone can serve as a proxy for estimating leptospiral loads shed in urine, and that shedding rates in urine are consistent over time. As obtaining urine is somewhat cumbersome, and some bladders are empty, our findings suggest that qPCR of kidney samples may be sufficient for inferring environmental loads, although these results should be confirmed for other reservoir hosts such as *R*. *rattus* [45]. The shedding of other species of bacteria, such as *Escherichia coli* [46] and *Coxiella burnetii* [43], has been shown to be highly variable over time in infected hosts in contrast to our results.

The information on urine shedding loads is critical for the construction of mathematical models predicting environmental contamination and consequently human disease risk [47,48]. The average loads in these paired samples ranged between 5.9 and 6.1 x 10^6^ GEq, but linear regression indicated that GEq in urine tended to be greater than that in kidney by a factor slightly less than 10. Our results are concordant with those obtained by experimentally infected rats where urine loads as high as 10^7^ genome equivalents (GEq) were found, as measured by qPCR and/or dark field microscopy [27,30]. The only other study reporting GEq in wild rats (*R*. *rattus*) found average loads in the kidney to be higher than what is reported here: 8.27x10^6^ (standard deviation of 4.72x10^6^). Corresponding urine loads were not evaluated in that study.

Multivariate analysis retained site of capture, L/W ratio (as a continuous variable), MAT titer and severity of wounds/scars as independent variables associated with leptospiral loads in both kidney and urine (Table 2). However, the significance of these variables differed between the sexes as discussed in greater detail below.

Our extrapolation indicates that the heterogeneity in leptospire shedding may be one of the major factors affecting environmental contamination by leptospires. Heterogeneity in the prevalence of leptospiral infection in rats has rarely been reported, almost certainly a reflection of small sample sizes, but was documented in Vancouver, Canada [18]. Geographical differences in leptospiral loads have not been previously reported by any study. The causes of these variations are unknown but could result from differences in the pathogen, environmental load, host genetics [44], or demographic characteristics. Most importantly, the consequences are also not known, but differences in leptospiral shedding and consequently in environmental contamination could be related to the spatial variation in human leptospirosis risk as evidenced in previous studies [49,50].

Leptospiral load in kidneys and urine was identical among male and female rats. When data were pooled, older animals, as determined by L/W ratio, tended to have higher kidney and urine loads. This association was stronger in males than in females indicating that heavier (and older) males had augmented leptospiral shedding (see also [45]). Reproductive status of females based on sexual maturity had no effect on bacterial loads, though limited sample size may be precluded this association.

When male and female data were pooled, increasing MAT titer was positively associated with kidney load. High MAT titers could reflect a high dose inoculum during infection and/or a short period after infection [51] either of which could lead to higher leptospiral kidney colonization and shedding. Of interest, while females had a similar positive association between MAT titer and kidney load to pooled rats, in males this association was negative for MAT = 50, albeit not significant. A similar pattern for urine load was observed for pooled, male and female rats, but again associations were not significant in males. Further studies are needed to elucidate the reasons why higher agglutination antibodies are related to higher leptospiral colonization in Norway rats.

The association between levels of wounding/scars with pathogen infection is well documented for Seoul virus infection among rats from Baltimore, and the presence of virus in rat saliva supports the potential for transmission via this route [52], but the observation here that increasing levels of wounds/scars were associated with increasing loads of leptospires (urine and kidney) was unexpected. Leptospires have been shown to be transmitted to guinea pigs via rat bite [53]. The load of leptospires in the saliva of wild rats has never been investigated, but as rats are constantly grooming (males spend up to 40% of the time in this activity [54]), it is highly plausible that saliva becomes contaminated with leptospires during oral grooming of the urogenital region-especially given the high bacterial loads in urine. Of note, there are documented instances of leptospiral transmission by rat bite to humans [53,55–57], suggesting that transmission through contaminated saliva occurs. This potential route for horizontal transmission among rats requires further investigation.

Based on our results, it is apparent that the routes of leptospiral transmission among Norway rats, or the inoculum doses required to achieve infection are still unknown, but whatever the route, high prevalence of infection were found among all age classes indicating efficient transmission. In experimental studies, the 50% colonization dose in Wistar rats was determined to be 10^4^ leptospires introduced intraperitoneally. However, in Golden Syrian hamsters, the LD_50_ is lower (<50 leptospires)[58]. Additionally, the development of leptospiral loads in the kidneys of another highly susceptible animal varied with the route of exposure [59].

This study was limited to two months in the dry season in a single year, precluding estimates of inter-year and inter-seasonal variations in leptospiral shedding. The demographic characteristics of rat populations are also likely to vary across seasons and years, such that of our example of a model Norway rat population and their contribution to leptospiral loads in the environment should be considered indicative rather than necessarily typical. Insufficient data are available on where rats are most abundant and whether the proximity of rats with higher loads of leptospiral shedding are most likely to lead to human exposure. However, in this study rats were captured close to human residences and our findings are relevant because there is a clear pattern of household clustering of persons infected by leptospires [60], implicating peri-domestic acquisition of infection. Sample sizes were low when stratified among the various covariates (eg sex, reproductive status, L/W ratio, site of capture), and therefore some associations may vary when reexamined with additional data. Of major importance, we were not able to distinguish between the differential contribution of live versus dead leptospires as identified by qPCR, possibly causing overestimation of the actual infectious leptospiral load present in kidneys and shed in the urine. We plan to conduct follow-up experiments to elucidate the fraction of live infectious leptospires using dark field microscopy or qPCR techniques that distinguish between living and dead leptospires.

Nonetheless, this study is unique as it is the first to address some of the critical, but unknown, parameters which can influence leptospiral infection, maintenance and shedding leading to environmental contamination. These factors directly influence the risk of leptospiral acquisition among humans and provide essential information on the epidemiological linkage between rats and humans.

## Supporting Information

S1 Table Demographic characteristics of the rat population stratified per geographic site, 2010. (DOCX)

S2 Table Estimates of *Leptospira* shedding considering rat demographic structure for the total and individual populations in Salvador, 2010. (DOCX)

S1 Fig Spiking experiment showing correlation between the number of spiked leptospires and the GEq detected by qPCR assay in urine and water. (TIF)
