# Supplementary material for: Patterns in Leptospira Shedding in Norway Rats (Rattus norvegicus) from Brazilian Slum Communities at High Risk of Disease Transmission
Source: PLoS Negl Trop Dis. 2015 Jun 5;9(6):e0003819. doi: 10.1371/journal.pntd.0003819 (PMC4457861; doi:10.1371/journal.pntd.0003819)
Supplement: S1 Table — (DOCX) [file pntd.0003819.s001.docx]

**S1 Table: Demographic characteristics of the rat population stratified per geographic site, 2010**

|  | PL1  (n=15) | 7A  (n=17) | PL6  (n=31) | PL8  (n=9) | P |
| --- | --- | --- | --- | --- | --- |
|  | No. (%) | | | |  |
| Sex |  |  |  |  |  |
| Male | 8 (53) | 8 (47) | 14 (43) | 4 (44) | NS |
| Weight category |  |  |  |  |  |
| Juvenile | 2 (14) | 3 (18) | 10 (32) | 0 (0) | NS |
| Sub-adult | 8 (53) | 11 (64) | 15 (48) | 4 (44) |  |
| Adult | 5 (33) | 3 (18) | 6 (19) | 5 (56) |  |
| Pregnant |  |  |  |  |  |
| Yes | 4 (57) | 3 (37) | 7 (39) | 3 (75) | NS |
|  |  |  |  |  |  |

NS: No significant
