# Supplementary material for: Patterns in Leptospira Shedding in Norway Rats (Rattus norvegicus) from Brazilian Slum Communities at High Risk of Disease Transmission
Source: PLoS Negl Trop Dis. 2015 Jun 5;9(6):e0003819. doi: 10.1371/journal.pntd.0003819 (PMC4457861; doi:10.1371/journal.pntd.0003819)
Supplement: S2 Table — (DOCX) [file pntd.0003819.s002.docx]

**S2 Table: Estimates of *Leptospira* shedding considering rat demographic structure for the total and individual populations in Salvador, 2010**

|  | No. of rats^1^ | Prevalence in urine^2^ | ml/ day^3^ | Log_10_ GEq/ml^4^ | Log_10_ Leptospires shed per day^5^ | Log_10_ Leptospires per m^2^ of soil /day |
| --- | --- | --- | --- | --- | --- | --- |
| Total | 82 |  |  |  | 9.1 | 5.0 |
| Juvenile | 16 | 0.77 | 15 | 5.6 |  |  |
| Sub-adult | 46 | 0.92 | 20 | 5.9 |  |  |
| Adult | 20 | 0.95 | 20 | 6 |  |  |
| Site PL1 | 15 |  |  |  | 10.6 | 6.5 |
| Juvenile | 1 | 1 | 15 | 6.7 |  |  |
| Sub-adult | 8 | 1 | 20 | 8.3 |  |  |
| Adult | 6 | 0.83 | 20 | 7.8 |  |  |
| Site PL2 | 15 |  |  |  | 9.6 | 5,6 |
| Juvenile | 3 | 1 | 15 | 6.7 |  |  |
| Sub-adult | 9 | 0.86 | 20 | 7.3 |  |  |
| Adult | 3 | 1 | 20 | 6.5 |  |  |
| Site PL6 | 6 |  |  |  | 9.6 | 5.5 |
| Juvenile | 1 | 1 | NA | 2.6 |  |  |
| Sub-adult | 4 | 0.75 | 20 | 7.7 |  |  |
| Adult | 1 | 1 | 20 | 7.6 |  |  |
| Site PL8 | 9 |  |  |  | 8,4 | 4.3 |
| Juvenile | 0 |  | 15 |  |  |  |
| Sub-adult | 5 | 0.5 | 20 | 2.2 |  |  |
| Adult | 4 | 0.75 | 20 | 6.6 |  |  |
| Site 7A | 37 |  |  |  | 9.3 | 5.2 |
| Juvenile | 11 | 0.5 | 15 | 4.6 |  |  |
| Sub-adult | 20 | 0.77 | 20 | 6.7 |  |  |
| Adult | 6 | 1 | 20 | 6.5 |  |  |
|  |  |  |  |  |  |  |

^1^ Number of rats in each mass/age class (NR.)

^2^ *Leptospira* prevalence in kidney (PREV: Table 1).

^3^ Volume (ml) of urine shed per 24 hours (VOL) as described by Donaldson [43].

^4^ Genomic equivalents of *Leptospira* per ml (LOAD: Table 1).

^5^ Based on density of rats (DENS) captured around households.
