# Supplementary figures and images for: Patterns in Leptospira Shedding in Norway Rats (Rattus norvegicus) from Brazilian Slum Communities at High Risk of Disease Transmission
Source: PLoS Negl Trop Dis. 2015 Jun 5;9(6):e0003819. doi: 10.1371/journal.pntd.0003819 (PMC4457861; doi:10.1371/journal.pntd.0003819)

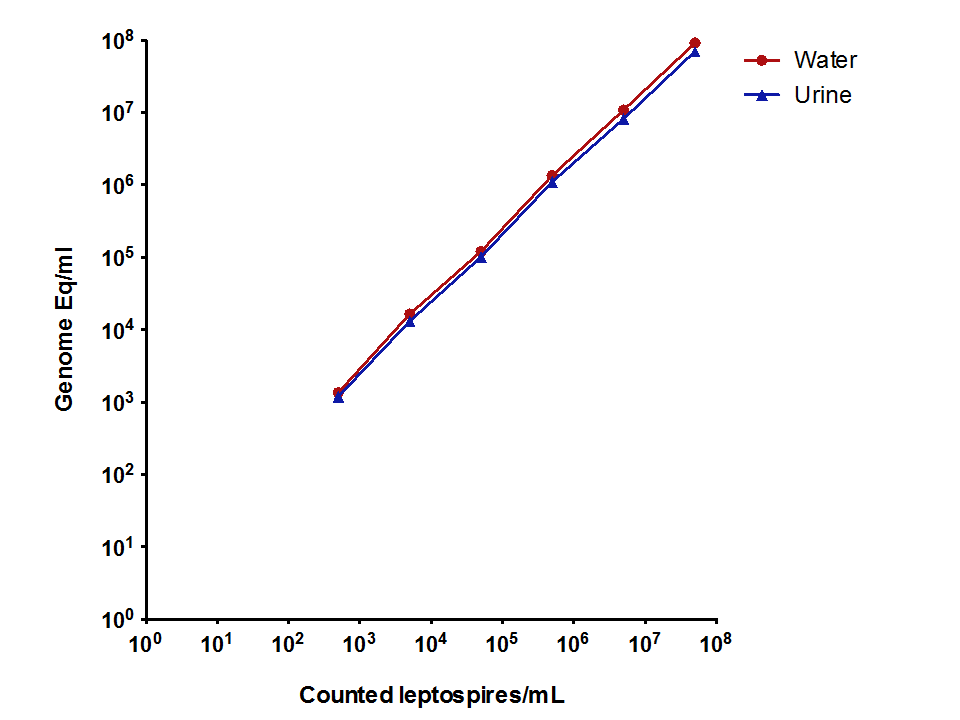

Supplement: S1 Fig — (TIF) [file pntd.0003819.s003.tif]
